# Integrating Large Language Models and Metaverse-Related Technologies in Urology Education: Viewpoint

**DOI:** 10.2196/93235

**Published:** 2026-10-19

**Authors:** Peizhe Li, Huijie Gong, Heyang Liu, Siyu Han, Yiqin Lu, Yongji Yan

**Affiliations:** 1 Department of Urology, Dongzhimen Hospital, Beijing University of Chinese Medicine, No. 5, Haiyuncang, Dongcheng District, Beijing, 100007, China, 86 84013150; 2 Department of Gynecology, Dongzhimen Hospital, Beijing University of Chinese Medicine, Beijing, China

**Keywords:** large language model, metaverse, artificial intelligence, AI, medical education, urology education

## Abstract

Urology education must prepare learners to integrate anatomical visualization, diagnostic reasoning, technical performance, communication, and ethical judgment, often under restricted working hours and limited access to high-fidelity cases. This viewpoint argues that large language models (LLMs) and metaverse-related technologies should be integrated as complementary, supervised educational tools rather than treated as autonomous replacements for educators or clinical experience. LLMs can support cognitive apprenticeship, documentation practice, assessment, and communication simulations, whereas extended reality, cloud computing, and virtual digital humans can support spatial, procedural, and remote learning. Our aim is to articulate 3 key messages: first, the educational value of these technologies depends on alignment with defined urological competencies; second, implementation should proceed in stages from governed data infrastructure to intelligent interaction and immersive multimodal training; and third, educational effectiveness must be established through prospective evaluations of transfer to clinical performance, not through technical novelty alone. We emphasize the need for rigorous randomized controlled trials, identify requirements for haptic and biomechanical fidelity, and discuss hallucination, bias, privacy, accountability, and deskilling. The responsible future of intelligent urology education will therefore require collaboration among learners, educators, clinicians, engineers, ethicists, institutions, and professional bodies.

## Introduction

### Educational Requirements in Urology

Training a urologist takes more than textbook recall. Residents need a working grasp of pelvic and retroperitoneal anatomy, have to read imaging, endoscopic findings, and pathology together, and must carry out precise operations. The work is also intimate and often distressing. Trainees have to counsel patients through oncological uncertainty, discuss loss of sexual or reproductive function, and manage stomas or urinary incontinence with tact.

However, clinical service alone rarely provides adequate, deliberate practice for these varied competencies. Duty-hour restrictions, patient safety obligations, and ethical limits on novice practice substantially narrow hands-on opportunities in the operating room and outpatient clinic [1]. The move toward advanced laparoscopy and robotic platforms adds a steep learning curve: fine bimanual control and spatial translation are hard to learn safely at the bedside [2]. The problem is clearest in uncommon or sensitive situations, such as penile cancer counseling, talks about sexual function, urinary diversion, fertility preservation, and disclosure of an uncertain cancer diagnosis [3].

### Aim and Viewpoint of This Article

This article is a viewpoint based on published work on AI, large language models (LLMs), virtual patients, extended reality (XR), robotic surgery, simulation, and medical education, together with our experience as urological and medical educators. We discuss 3 key points. First, LLMs and metaverse-related technologies cover different but linked parts of training; LLMs can support cognitive and communicative learning, while XR and related systems can support spatial and procedural learning [4]. Second, these tools should be brought in through a staged, competency-based, and human-supervised framework. Third, claims of educational benefit should rest on validated measures and evidence of transfer to clinical performance, not on realism, usability, or model accuracy alone.

## Cognitive and Communicative Learning With LLMs

### General-Purpose and Specialized LLMs

General-purpose LLMs can explain concepts, generate questions, summarize literature, role-play clinical conversations, and provide feedback on written reasoning [5,6]. Their flexibility makes them useful for early exploratory learning [6,7]. However, a general-purpose model may lack reliable knowledge of local workflows, specialty terminology, guideline updates, or the clinical context required for urological decision-making. It may also produce fluent but unsupported answers.

A specialized or retrieval-augmented model can be limited to curated guidelines, institutional protocols, checked teaching materials, and properly deidentified cases [8]. Fine-tuning or retrieval augmentation may improve specialty relevance, traceability, and consistency, but neither approach guarantees correctness [9]. A tuned model can reproduce errors or biases present in its source data, and a retrieval system can return an outdated, incomplete, or poorly matched document. Accordingly, educators should compare general-purpose and specialized models against an expert-annotated reference set before deployment and should display source provenance, uncertainty, and the date of guideline retrieval whenever feasible [8,10].

### Uses in Assessment and Clinical Reasoning

LLMs may assist, but should not independently determine, several components of urological assessment. Potential applications include generating formative questions; evaluating the completeness of a history or operative note; identifying whether a learner considered relevant differential diagnoses; providing structured feedback on documentation; and analyzing simulated consultations for missed questions, unclear explanations, or inappropriate language [11-13]. They may also support oral examinations by presenting sequential information, such as a patient with gross hematuria whose history, urinalysis, imaging, cystoscopy, and pathology findings are released progressively.

In urological oncology, an educational system should teach diagnostic algorithms rather than merely provide final answers. For example, a hematuria case may require risk assessment, urine testing, imaging selection, cystoscopic evaluation, and interpretation of pathology. A prostate cancer case may require integration of prostate-specific antigen, digital rectal examination, multiparametric magnetic resonance imaging, targeted or systematic biopsy, grade group, staging, and patient preferences. A renal mass case may require consideration of imaging phenotype, renal function, tumor complexity, metastatic evaluation, and the trade-off between oncological control and renal preservation. These pathways should be represented as branching decisions with explicit uncertainty and should be linked to expert-verified guidelines and contemporary diagnostic literature [14-16].

The appropriate educational role of an LLM is therefore to make reasoning visible and discussable. It can ask why a learner selected a test, identify missing information, compare plausible options, and prompt reflection. It should not silently replace the learner’s reasoning or present one recommendation as universally correct. Representative LLMs illustrate the range of possible applications, from clinical knowledge reasoning and electronic health record processing to virtual patient simulation and guideline-based tutoring (Table 1).

**Table 1.** Representative large language models and their potential applications in urology education.

Models	Developer/researcher	Key features	Potential applications in urology education
Med-PaLM 2	Google Research [9]	Clinical knowledge reasoning and performance on USMLE-style^a^ evaluations	Generates complex decision-making questions and serves as a virtual examiner for advanced resident training
GatorTron	University of Florida [17]	Trained on large volumes of EHRs^b^ and designed to process clinical narrative language	Simulates urological clinic dialogues and medical records for interview and documentation training
AIPatient	Yu et al [18]	EHR-based knowledge graphs and retrieval-augmented generation for virtual patient interviewing	Provides high-fidelity practice for breaking bad news and preoperative communication, with performance assessment
BioMedLM	Stanford University and MosaicML [19]	Biomedical literature comprehension and generation	Supports literature review and research skills development on urological topics
UroBot	Hetz et al [20]	Urology question answering enhanced with EAU^c^ guidelines	Provides guideline-based tutoring and knowledge tracing

a USMLE: United States Medical Licensing Examination.

b EHR: electronic health record.

c EAU: European Association of Urology.

### Hallucination, Empathy, and Human Supervision

LLM errors are not limited to isolated factual mistakes. They may include input-related errors caused by ambiguous, incomplete, or incorrectly transcribed data; general knowledge errors in which the model produces an inaccurate medical statement; retrieval-related errors caused by irrelevant or outdated sources; reasoning errors in which correct facts are combined incorrectly; and communication errors in which an emotionally inappropriate response is phrased fluently [10]. In a sensitive consultation, an apparently empathic sentence may still be clinically misleading, culturally inappropriate, or insufficiently responsive to the patient’s distress.

These limits matter most when an LLM is used to simulate a patient with cancer or sexual health concerns. The system should be restricted to teaching, clearly labeled as simulated, watched by an educator, and blocked from unsupervised clinical advice. Feedback to trainees should cover not only facts, but also empathy, privacy, shared decision-making, and psychosocial needs. The instructor still has to review the scenario, correct errors, and lead the debrief [21-24].

## Immersive, Spatial, and Procedural Learning

### XR and Surgical Simulation

The educational value of virtual reality, augmented reality, and mixed reality lies in repeated practice, controlled variation of difficulty, spatial visualization, and the possibility of objective data capture [4,25]. Virtual reality may support laparoscopic, endoscopic, and robotic skills, while augmented reality and mixed reality can overlay patient-specific anatomy or imaging information onto a physical environment. Studies of urological simulation have shown gains in selected technical tasks, including transurethral procedures, robotic needle passing, and endoscopic spatial orientation [26-30]. These findings support XR as a training method. However, XR does not, by itself, show shorter operative time, fewer complications, better oncological results, or lasting transfer to independent clinical work.

### Haptic and Biomechanical Fidelity

A credible surgical simulator must address more than visual realism. Laparoscopic and robotic skills also depend on force perception, instrument-tissue interaction, tissue deformation, traction, cutting resistance, needle passage, and recognition of abnormal structures. Urological tissues also differ in elasticity, vascularity, fragility, and response to manipulation. A simulator that provides visually appealing anatomy, but unrealistic resistance, may teach inappropriate force application [31].

Technical needs include real-time tissue deformation models, collision detection, instrument friction, variable tissue stiffness, bleeding and smoke, camera-control limits, and latency low enough for force feedback. Haptic systems should be calibrated against expert-defined task requirements and, where possible, against physical or instrumented reference data. Higher fidelity is not always better. The level of fidelity should match the skill being taught, the learner’s stage, safety, cost, and evidence of benefit. Future validation studies should report haptic fidelity apart from visual fidelity and test whether each one improves measured performance.

### Remote Learning and Virtual Digital Humans

Cloud computing can take on heavy rendering, storage, analytics, and remote access. Remote and virtual surgical teaching may widen access to expert instruction, especially where faculty or equipment are scarce [32-35]. Virtual digital humans can provide an embodied interface for LLM-based patients, mentors, or examiners, combining dialogue with gestures, facial expressions, anatomical models, and scenario-specific behavior [36,37]. Their educational benefit will depend on the accuracy of their clinical content, the appropriateness of their affective responses, the quality of their multimodal interaction, and the extent to which learners perceive the scenarios as educationally authentic.

A further concern is that highly repeatable and technically perfect AI-generated cases may be valued less than real cases containing ambiguity, workflow interruptions, competing priorities, and ethically difficult decisions. Case systems should therefore include controlled variability and realistic clinical dilemmas rather than presenting idealized pathways only [38].

## A Staged Framework With a Clinical Example

### Data and Governance Foundation

We propose a 3-stage implementation framework that moves from a governed data and infrastructure foundation to intelligent interaction and then immersive training. The framework is summarized in Figure 1. The first stage is the creation of a governed teaching-data infrastructure. Relevant materials may include deidentified histories, laboratory results, imaging, endoscopy, pathology, operative videos, and assessment rubrics. Data should be minimized, access controlled, documented, and reviewed for representativeness. Educational datasets should not be assembled from electronic health records without appropriate institutional approval, deidentification, data use agreements, and safeguards against reidentification [8,10,17,39].

**Figure 1.**
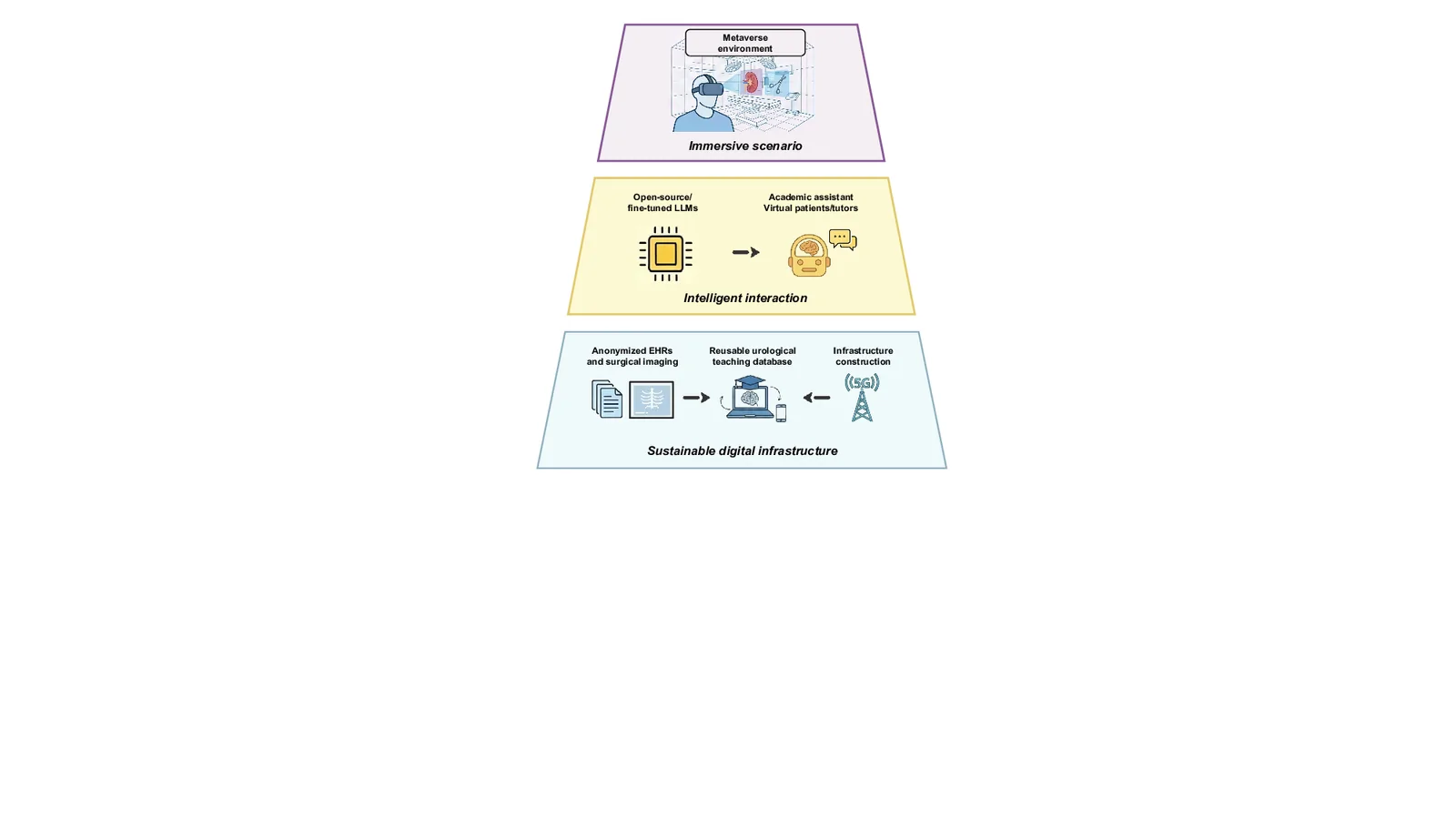
A framework for the synergistic integration of large language models and the metaverse in urology education. EHR: electronic health record; LLM: large language model.

### Intelligent Interaction

The second stage is the development of specialty-aware agents that retrieve approved content, present cases progressively, ask targeted questions, and provide explainable feedback. Agents should record the information used to generate an answer, distinguish evidence from inference, and escalate uncertain or unsafe outputs to a human educator. General-purpose LLMs may be used for low-risk brainstorming or language practice, whereas higher-risk diagnostic and assessment tasks should use constrained systems validated against expert standards.

### Immersive Multimodal Training

The third stage places these agents in XR environments. Consider a simulated patient with visible hematuria. The learner first takes a history, looks for risk factors and red flags, explains the initial workup, reviews imaging, interprets cystoscopic findings, and discusses the pathology report. The LLM-based patient can show anxiety and ask questions, while the XR environment shows anatomy and procedural steps. The educator can then review the diagnostic sequence, communication, time use, unnecessary tests, and technical performance. A similar prostate cancer case may combine magnetic resonance imaging interpretation, biopsy counseling, staging, treatment discussion, and concerns about sexual function. The aim is not a perfect virtual world; it is to bring cognitive, technical, and humanistic skills together in a setting that is safe and can be assessed [4,26,37].

## Proposed Evaluation and Implementation Safeguards

### Prospective Randomized Controlled Trials

Rigorous, well-designed randomized controlled trials are essential to establish whether LLM- and metaverse-supported educational tools improve urology training beyond conventional approaches. Future studies should prioritize clinically meaningful and validated outcomes rather than technological novelty, learner satisfaction, or simulator scores alone. Relevant domains include technical performance, diagnostic reasoning, communication, retention, and, critically, transfer of learning to supervised clinical practice. Outcome selection should reflect the specific competency targeted by the intervention and should be informed by established work on diagnostic complexity and objective assessment in XR [31]. Confidence in such evidence will also depend on sufficiently independent assessment and appropriate consideration of variation in learner experience and training context. Equally important, evaluation should examine the durability, generalizability, and potential unintended educational effects of these tools. Evidence of improved performance within a simulated environment, although valuable, should not be considered sufficient to demonstrate clinical competence or justify broad educational implementation.

### Ethical, Logistical, and Equity Considerations

LLM and metaverse systems can be computationally expensive, which raises costs and may widen gaps between institutions [40,41]. Sensitive medical data require governance, encryption, auditing, retention limits, and clear responsibility for errors. Biased training data may produce unequal performance across sex, age, ethnicity, language, disability, or socioeconomic groups [39]. Systems should therefore undergo subgroup validation and continuous monitoring.

Overreliance may also reduce independent reasoning or encourage deskilling [24]. Curricula should require learners to make an initial independent assessment before viewing AI suggestions, explain disagreements with the system, and complete periodic no-AI assessments. Human educators should approve cases, supervise use, evaluate performance, and remain accountable for educational decisions.

## Conclusions

### Key Messages

LLMs and metaverse-related technologies may strengthen urology education when their roles are clearly differentiated and deliberately integrated. LLMs are most useful for cognitive apprenticeship, documentation, formative assessment, and supervised communication practice. XR, haptics, cloud platforms, and virtual digital humans are most useful for spatial, procedural, and remote learning. Neither technological fluency nor visual realism is equivalent to competence.

### Future Directions

The field should move from speculative demonstrations to competency-based implementation and prospective validation. The next generation of studies should report transparent model behavior, haptic and biomechanical fidelity, subgroup performance, educational authenticity, retention, and transfer to clinical practice. A staged, equitable, and human-supervised approach offers a more defensible path toward intelligent urology education than the assumption that automation or immersion will inevitably improve training.

## Abbreviations

LLM: large language model
XR: extended reality
